# Ruptured urinary bladder attributable to urethral compression by a haematoma after vertebral fracture in a bull

**DOI:** 10.1186/1751-0147-56-17

**Published:** 2014-03-26

**Authors:** Ueli Braun, Luzia Trösch, Titus Sydler

**Affiliations:** 1 Department of Farm Animals, Vetsuisse Faculty, University of Zurich, Zurich, Switzerland; 2 Institute of Veterinary Pathology, Vetsuisse Faculty, University of Zurich, Zurich, Switzerland

## Abstract

**Background:**

In male cattle, rupture of the urinary bladder is usually associated with urethral obstruction by uroliths. Less common causes include urethral compression or stricture. This case report describes the findings in a young Limousion breeding bull with rupture of the urinary bladder because of urethral compression by a haematoma after coccygeal fracture.

**Case presentation:**

The bull had been introduced into a 40-head Red-Holstein herd one week before being injured. One week after introduction to the herd, the bull had an acute onset of anorexia and he was referred to the clinic. There was marked abdominal distension, reduced skin turgor and enophthalmus. The serum concentration of urea and creatinine was increased. Ultrasonographic examination revealed severe ascites and abdominocentesis yielded clear yellow fluid with high urea and creatinine concentrations, which supported a diagnosis of uroperitoneum. The bull was euthanatized because of a poor prognosis. Postmortem examination revealed a comminuted fracture of the first two coccygeal vertebrae associated with a massive haematoma that obstructed entire pelvic cavity. The haematoma compressed the urethra thereby preventing outflow of urine, which resulted in a 5-cm tear ventrally at the neck of the bladder. It was assumed that the newly-introduced bull had sustained the vertebral fractures when he was mounted by a cow.

**Conclusions:**

The present case study serves to expand the differential diagnosis of urinary bladder rupture. Therefore, in addition to obstructive urolithiasis, compression and stricture of the urethra might be considered in male cattle with uroperitoneum.

## Background

Rupture of the urinary bladder is usually associated with dystocia in cows [1,2], and urinary tract disorders [3,4], particularly urethral obstruction by uroliths, are the leading cause in male cattle. Less common causes of bladder rupture include urethral compression or stricture [3], which are usually attributable to injury, necrotic inflammation or scar tissue formation after surgery (urethrotomy). Bladder rupture is rarely caused by compression of the urethra by tumours, abscesses or haematomas [3]. Unless promptly diagnosed and treated, obstruction results in rupture of the urethra or urinary bladder with subsequent uroperitoneum, which is characterised clinically by marked abdominal distension. The present case report describes rupture of the urinary bladder attributable to urethral compression by a haematoma after vertebral fracture in a young bull.

## Case presentation

The patient was a 1.3-year-old Limousin bull that had been introduced into a 40-head Red-Holstein herd one week before being injured. The herd was housed in a free stall barn with access to a courtyard as well as pasture during the day. One week after introduction to the herd, the bull had an acute onset of anorexia and disturbed general condition. Clinical examination by the referring veterinarian revealed abdominal distension and positive swinging and percussion auscultation of the abdomen on the left side, which was interpreted as left displacement of the abomasum. The bull was referred to the Department of Farm Animals, University of Zurich. On admission, the general condition and behavior of the bull were disturbed and there was reduced skin turgor, enophthalmus and scleral injection. The rectal temperature was normal at 38.7°C, the heart rate was increased at 96 beats per minute and there was marked abdominal distension. The rumen was atonic and distended, and swinging and percussion auscultation were positive on the left side of the abdomen. Intestinal motility was reduced and the abdominal wall was tense. Transrectal examination of the abdomen was not possible because the pelvic cavity was severely constricted. There was a reduced amount of faeces, which were olive coloured and porridge-like in consistency. A urine sample could not be collected.

The results of haematological examination showed haemoconcentration with a haematocrit of 54% (normal 30 – 35%), leukocytosis with neutrophilia (total leukocyte count of 16’900 leukocytes/μl blood; normal 5’000 – 10’000 leukocytes/μl), hyperproteinaemia with a total plasma protein concentration of 106 g/l, normal 60 – 80 g/l) and hyperfibrinogenaemia (fibrinogen concentration 8 g/l, normal 4 – 5 g/l). The serum concentration of urea (46 mmol/l, normal 2.4 – 6.5 mmol/) and creatinine (2’226 μmol/l, normal 80 – 120 μmol/l) were increased. The serum concentrations of sodium, potassium and chloride were lower than normal and that of inorganic phosphorus was increased (sodium 130 mmol/l, normal 145 – 155 mmol/l; potassium 3.6 mmol/l, normal 4 – 5 mmol/l; chloride 57 mmol/l, normal 95 – 105 mmol/l; inorganic phosphorus 4.03 mmol/l, normal 1.3 – 2.4 mmol/l). The results of blood gas analysis revealed a compensated metabolic alkalosis with a base excess of +7.7 mmol/l (normal -2 to +2 mmol/l) and a blood pH of 7.41 (normal 7.40 – 7.50).

Ultrasonographic examination revealed severe ascites involving the entire abdomen. The fluid appeared homogeneous and anechoic, and abdominocentesis yielded clear yellow fluid that smelled of urine. The urea and creatinine concentrations were 57 mmol/l and 7’042 μmol/l, respectively, which supported a diagnosis of uroperitoneum.

Based on all the findings, a diagnosis of uroperitoneum caused by urinary bladder rupture was made. The cause was thought to be obstructive urolithiasis. The mass causing pelvic obstruction could not be interpreted, but rule-outs included inflammation, tumour, abscess and haematoma. Because of a poor prognosis, the bull was euthanatized and underwent postmortem examination. This revealed a comminuted fracture of the first two coccygeal vertebrae associated with a massive haematoma that obstructed the entire pelvic cavity. The haematoma compressed the urethra thereby preventing outflow of urine, which resulted in a 5-cm tear ventrally at the neck of the bladder. The final diagnosis was rupture of the urinary bladder because of urethral compression by a haematoma after coccygeal vertebral fracture.

Inability to defecate has been reported as a complication of fracture of the proximal coccygeal vertebrae [5], but to our knowledge, the complication encountered in our patient has not been described. The common cause of the fracture in this young, relatively small and newly-introduced bull was mounting by a cow because this is the most likely common cause of coccygeal vertebral fractures in cattle [6]. Our clinical findings were in agreement with other reports of cattle with urinary bladder rupture and uroperitoneum [3,4,7,8]. The main sign was abdominal distension with progressive deterioration in general condition. Haemoconcentration, azotaemia, hypochloraemia and hyperphosphataemia were also characteristic of uroperitoneum [9,10].

Urine osmolality is two to three times higher than the osmolality of interstitial fluid [9]. The osmotic gradient created by uroperitoneum resulted in fluid leaving the vascular space and entering the abdominal cavity, which resulted in haemoconcentration. Hyponatraemia and hypochloridaemia were attributable to the concentration gradient created by the lower concentrations of sodium and chloride in urine compared with blood [9]. Urea and creatinine concentrations are higher in urine and the resultant gradient favoured diffusion of these nitrogenous wastes products into the blood causing azotaemia [9]. Hyperphosphataemia was due to decreased glomerular filtration rate [10]. Uroperitoneum is commonly associated with metabolic alkalosis in cattle, whereas foals and dogs with a ruptured bladder usually have metabolic acidosis [10].

The most common cause of urinary bladder rupture in male cattle is obstructive urolithiasis. In cows, uroperitoneum due to urinary bladder rupture is most often associated with dystocia [1,2,11], but may also be the result of a breeding injury, traumatic catheterisation, accidental introduction of an insemination pipette into the urinary bladder or sadism [3]. Haematomas in the pelvic region may also result from haemorrhage into the broad ligament or mesentery because of dystocia or from haemorrhage within the renal capsule after kidney trauma [12]. Uroperitoneum attributable to rupture of the urachus has also been described in cows [13,14]. However, to the authors‘ knowledge, haematoma formation with subsequent compression of the urethra after coccygeal fracture has not been described.

## Conclusion

The present case study serves to expand the differential diagnosis of urinary bladder rupture. Therefore, in addition to obstructive urolithiasis, compression and stricture of the urethra might be considered in male cattle with uroperitoneum.

## Competing interests

The authors declare that they have no competing interests.

## Author’s contributions

UB prepared the manuscript and supervised the clinical examination, LT examined the bull, TS performed the postmortem examination. All authors have read and approved the manuscript.
